# Synthesis and Biological Evaluation of New Thiosemicarbazone Derivative Schiff Bases as Monoamine Oxidase Inhibitory Agents

**DOI:** 10.3390/molecules23010060

**Published:** 2017-12-28

**Authors:** Betül Kaya Çavuşoğlu, Begüm Nurpelin Sağlık, Derya Osmaniye, Serkan Levent, Ulviye Acar Çevik, Abdullah Burak Karaduman, Yusuf Özkay, Zafer Asım Kaplancıklı

**Affiliations:** 1 Department of Pharmaceutical Chemistry, Faculty of Pharmacy, Anadolu University, 26470 Eskişehir, Turkey; betulkaya@anadolu.edu.tr (B.K.Ç.); bnsaglik@anadolu.edu.tr (B.N.S.); dosmaniye@anadolu.edu.tr (D.O.); serkanlevent@anadolu.edu.tr (S.L.); uacar@anadolu.edu.tr (U.A.Ç.); zakaplan@anadolu.edu.tr (Z.A.K.); 2 Doping and Narcotic Compounds Analysis Laboratory, Faculty of Pharmacy, Anadolu University, 26470 Eskişehir, Turkey; 3 Department of Pharmaceutical Toxicology, Faculty of Pharmacy, Anadolu University, 26470 Eskişehir, Turkey; abkaraduman@anadolu.edu.tr

**Keywords:** thiosemicarbazone, MAO-A, MAO-B, docking, MTT, enzyme kinetic study, ADME

## Abstract

Twenty-six novel thiosemicarbazone derivative **B1**–**B26** were synthesized via condensation reactions between the corresponding thiosemicarbazides and aldehydes. The chemical characterization of the compounds was carried out by infrared (IR), mass (MS), proton and carbon nuclear magnetic resonance (^1^H- and ^13^C-NMR) spectroscopic analyses. The compounds were investigated for their monoamine oxidase A (MAO-A) and monoamine oxidase B (MAO-B) inhibitory activity and most of them were more potent against MAO-A enzyme when compared with MAO-B enzyme. *N*-Cyclohexyl-2-[4-[(4-chlorophenyl)thio]benzylidene]hydrazine-1-carbothioamide (**B24**) was the most active compound against MAO-A. The enzyme kinetics study revealed that compound **B24** has a reversible and competitive mode of binding. Interaction modes between compound **B24** and MAO-A were clarified by docking studies. In addition, the favourable absorption, distribution, metabolism, and excretion (ADME) properties and non-toxic nature of compound **B24** make this compound a promising MAO-A inhibitor.

## 1. Introduction

The process of synaptic neurotransmission is ensured by the correct functioning of enzymes. Monoamine oxidases (MAOs) are flavoproteins bound to the outer membranes of mitochondria throughout the brain, catalysing the oxidative deamination of monoamine neurotransmitters, thereby participating in this mechanism by modulating the levels of neurotransmitters [1,2]. Two subtypes of MAO exist in mammals, namely MAO-A and MAO-B, varying in substrate specificity, sensitivity to inhibitors and amino acid sequences [3,4]. MAO-A preferentially oxidizes norepinephrine and serotonin and is selectively inhibited by clorgyline, while MAO-B preferentially deaminates dopamine and is irreversibly inhibited by l-deprenyl [5,6]. Both isoforms are significant drug targets in the therapy of neuropsychiatric and neurodegenerative disorders. Inhibitors of MAO-A are employed as antidepressants and anxiolytics [7,8] while MAO-B inhibitors are clinically used for individuals suffering from Parkinson’s disease and Alzheimer’s disease [9]. Adverse effects of irreversible and nonselective MAO inhibitors [10] have led to great interest in the discovery of novel and selective drug candidates.

Excessive levels of metal ions accumulate in brain with aging, leading to neurodegeneration. Metal-induced neurotoxicity is assumed to be connected with several neurological diseases [11]. In recent years, chelation therapy has become a useful treatment for the symptoms associated with the central nervous system [12]. Thiosemicarbazones (TSCs) are a class of sulphur- and nitrogen- containing compounds that are able to form complexes with metal ions present in biological systems [13]. Due to the strong reactivity of the hydrazine nitrogen (C=N) and thioketone (C=S) groups, TCS compounds represent a versatile class of Schiff bases and are important in medicinal chemistry [14]. Recently, there has been a concentrated research on Schiff bases and their complexes due to their range of biological activity including antifungal, antibacterial, antimalarial, antiviral, anticancer, anti-inflammatory, sedative-hypnotic, antidepressant, analgesic, cytotoxic, anticonvulsant [15,16,17,18,19,20,21,22].

TSCs are small molecules that can cross the blood brain barrier and hence be used as potential drugs in the treatment of neurodegenerative diseases. Furthermore, it has been reported that TSCs have some common features as H-bond acceptors, H-bond donors, hydrophobic substructures, positive ionizable atoms, and aromatic rings to interact with MAO enzymes [23].

Taking these facts into account, in this study, we prepared a series of thiosemicarbazide derivatives, which were substituted with a various aliphatic and aromatic groups at the N(1) and N(4) atoms. The newly synthesized compounds were examined for their inhibitory activities against MAO-A and MAO-B. Enzyme kinetic and docking studies were also carried out to clarify their inhibition profiles and mechanism(s) of action.

## 2. Results and Discussion

### 2.1. Chemistry

**Scheme 1:**
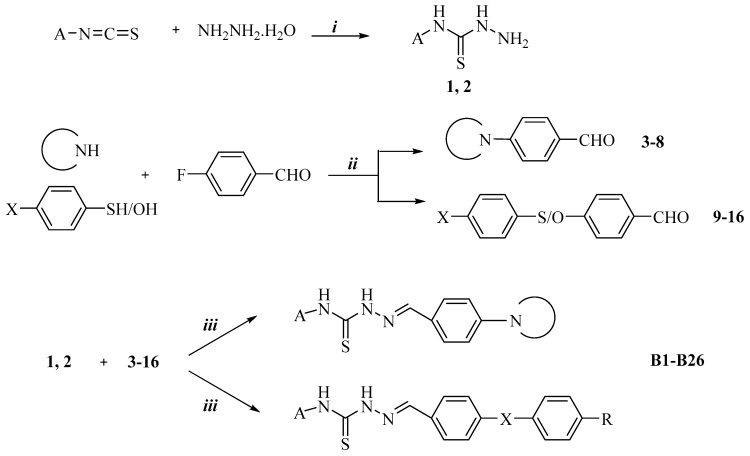
The synthetic route of the compounds **B1**–**B26**. Reagents and conditions: (**i**) ethanol, ice bath, 2 h; (**ii**) *N,N*-dimethylformamide (DMF), K_2_CO_3_, reflux, 24 h; (**iii**) ethanol, reflux, 4–10 h.

The synthetic pathways and structural features of the compounds **B1**–**B26** are outlined in Scheme 1. In the first step, phenyl- or cyclohexylisothiocyanate and hydrazine hydrate were reacted to obtain *N*-phenyl/cyclohexylhydrazinecarbothioamides **1**,**2**. In the second step, in order to synthesize aldehyde derivatives **3**–**16**, suitable secondary amines or 4-substituted phenol/thiophenols and 4-fluorophenylbenzaldehyde were refluxed in dimethylformamide. In the final step, the condensation in ethanol of *N*-phenyl/cyclohexylhydrazinecarbothioamides **1**,**2** and the synthesized 4-substituted benzaldehyde derivatives **3**–**16** gave the title compounds. 

The structures of all final compounds were elucidated by infrared, mass, ^1^H- and ^13^C-NMR spectroscopy experiments. In the infrared (IR) spectra, N-H, aromatic and aromatic C-Hs, C=N, C=C, C-N and C-O bands were observed. N-H bands were recorded around 3280 cm^−1^. C-H bands for aromatic and aliphatic examples were observed between 3157 cm^−1^ and 2929 cm^−1^. C=N and C=C groups were recorded at 1600 cm^−1^–1442 cm^−1^. C-N and C-O bands were observed between 1287–1124 cm^−1^. In the ^1^H-NMR spectra, alkyl group protons had peaks between 0.92 ppm and 4.37 ppm. The chemical shift values of aromatic ring protons were between 6.81 ppm and 7.82 ppm, depending on the substituent groups. Imine protons appeared between 7.83 ppm and 8.14 ppm, while N-H protons were seen at 7.91 ppm−11.85 ppm. In the ^13^C-NMR spectra, aliphatic and aromatic carbons were observed between 19.65 ppm−55.90 ppm and 112.14 ppm−163.11 ppm, respectively. Thiocarbonyl carbons were observed between 175.53 ppm and 176.44 ppm. In High Resolution Mass (HRMS) analysis, the calculated and expected masses were matched at the ppm level. The IR, ^1^H-NMR, ^13^C-NMR, and HRMS spectra of compound **B24** are presented in supplementary file.

### 2.2. Monoamine oxidase Inhibition Assay

**Figure 1:**
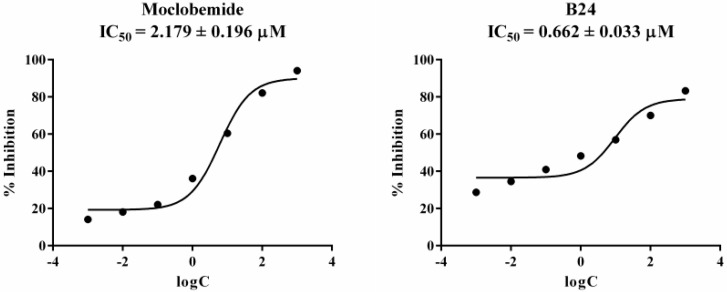
Further screening to determine inhibition potency and a half-maximal inhibitory concentration (IC_50_) values of **B24** and moclobemide against MAO-A.

The novel series of thiosemicarbazone derivatives **B1**–**B26** were investigated for their MAO-A and MAO-B inhibitory activity by a two-step fluorometric method. In the first step, compounds **B1**–**B26** were screened at 10^−3^ and 10^−4^ M concentrations. Compounds that indicate more than 50% inhibition at first step, were tested at 10^−5^–10^−9^ M concentrations. As seen in Table 1, compounds **B19**, **B21**–**B26** showed 50% inhibitory activity against MAO-A at 10^−3^ concentration. Except for compound **B24**, these compounds did not display 50% inhibition at 10^−4^ concentration, thus only compound **B24** was selected for the second step activity assay. The result of this step is presented in Figure 1. The second screening of compound **B24** against MAO-A revealed that this compound displayed a half-maximal inhibitory concentration (IC_50_) of 0.662 μM, whereas reference drug moclobemide had an IC_50_ of 2.179 μM. None of the compounds showed inhibitory effect towards MAO-B at tested concentrations. These findings suggest that compounds are more selective inhibitors against MAO-A than MAO-B. 

Generally, compounds **B14**–**B26** have been found to more active than compounds **B1**–**B13**. Therefore, it can be concluded that a cyclohexyl ring at the N(1) position is more favourable for the MAO-A inhibitory activity. Furthermore, between compounds **B14**–**B26**, compounds containing 4-substituted phenyl ring linked by an oxygen or sulphur atom were indicated as more potent. As the most active inhibitory agent is *N*-cyclohexyl-2-[4-[(4-chlorophenyl)thio]benzylidene]hydrazine-1-carbothioamide (**B24**), a 4-chlorophenyl group linked with a sulphur atom was demonstrated to be beneficial for MAO-A inhibitory activity.

### 2.3. Enzyme Kinetic Studies

**Figure 2:**
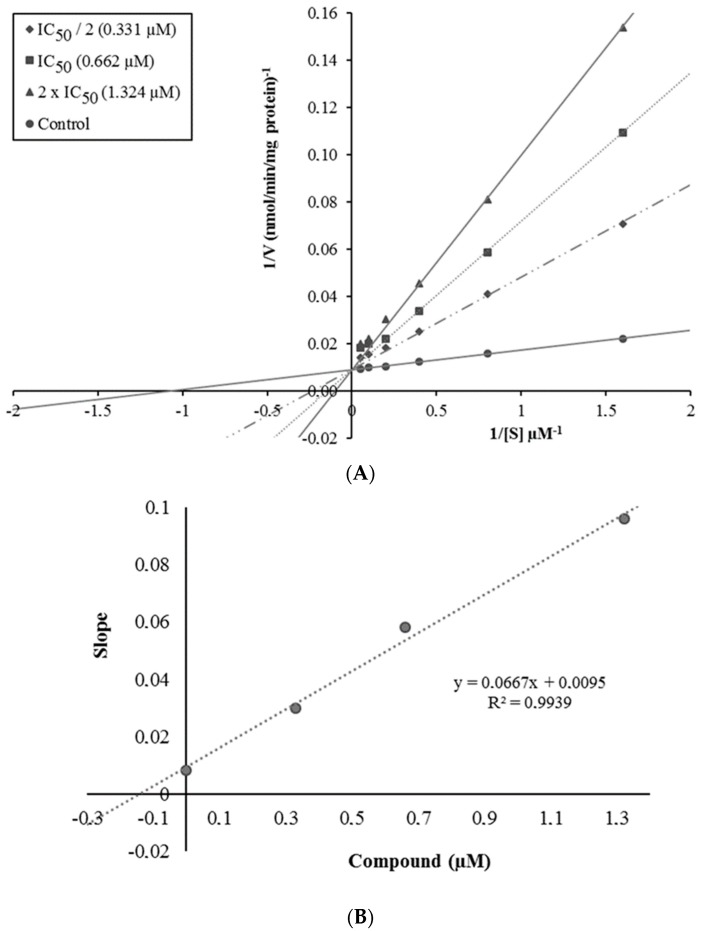
(**A**) Lineweaver–Burk plots for the inhibition of MAO-A by compound **B24**. [S], substrate concentration (μM); V, reaction velocity (nmol/min/mg protein). Inhibitor concentrations are shown at the left. Michaelis-Menten constant (K_m_) values from IC_50_/2 to control; 3.303, 6.455, 10.656 and 0.933 (μM). V_max_ value of the competitive inhibition; 111.015 ± 2.557 (nmol/min/mg protein); (**B**) Secondary plot for calculation of steady-state inhibition constant (K_i_) of compound **B24**. K_i_ was calculated as 0.142 μM.

The mechanism of MAO-A inhibition was examined by enzyme kinetics, following a similar procedure to the MAO inhibition assay. The linear Lineweaver-Burk graphics were used to determine the type of inhibition. Enzyme kinetics were analyzed by recording substrate velocity curves in the absence and presence of the most potent compound **B24**, which was prepared at IC_50_/2, IC_50_ and 2 × IC_50_ concentrations. The initial velocity measurements were gained at different substrate (tyramine) concentrations ranging from 20 µM to 0.625 µM. The steady-state inhibition constant (K_i_) values of compound **B24** was determined from the secondary plot slope versus varying concentrations. The graphical analysis of steady-state inhibition data for compound **B24** is presented in Figure 2.

Enzyme inhibitors can be classified as reversible or irreversible relative to the type of interaction. Uncompetitive, competitive, noncompetitive, and mixed inhibitions are the subtypes of reversible inhibitors than can be determined by Lineweaver-Burk graphics [24]. It is known that uncompetitive type inhibition is observed with a graph, including the parallel lines of inhibitor and control without any cross. If there is a line crossing at the same point but not on the x-axis or y-axis the inhibition type is called mixed-type. Non-competitive inhibitors display the same intercept on the x-axis but with diverse slopes and intercepts on the y-axis between the two data sets. Oppositely, competitive inhibitors give plots with the same intercept on the y-axis but different slopes and intercepts on the x-axis as observed in Figure 2. Therefore, it can be stated that compound **B24** is a reversible and competitive inhibitor, showing similar inhibition features as the substrate. The K_i_ value for compound **B24** was calculated as 0.142 μM for the inhibition of MAO-A.

### 2.4. Cytotoxicity Test

After the preliminary enzyme inhibition assay, compound **B24** was selected for the second step, and besides its toxicity was measured because the cytotoxicity of a compound is a crucial parameter in medicinal chemistry to obtain a safe drug candidate. A methylthiazolyl tetrazolium (MTT) assay [25] was applied to screen the cytotoxic effects of compound **B24** on healthy NIH/3T3 mouse embryonic fibroblast cell lines (ATCC CRL1658, London, UK). It has been determined that compound **B24** had an IC_50_ value of 24.6 µM against NIH/3T3 cells, while its IC_50_ value was 0.662 µM against MAO-A, thus proving that compound **B24** is non-toxic at its effective concentration towards MAO-A.

### 2.5. Molecular Docking Studies

**Figure 3:**
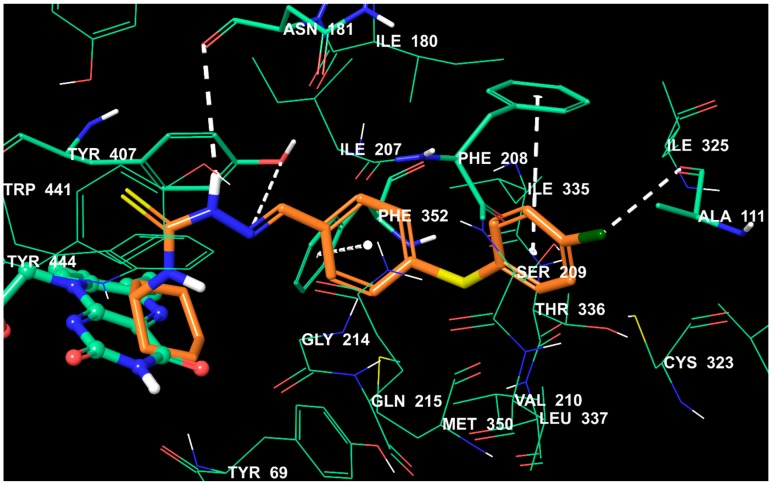
The interacting mode of compound **B24** in the active region of MAO-A. The inhibitor and the important residues in the active site of the enzyme are presented by tube model. The flavin adenine dinucleotide (FAD) molecule is colored green with ball and stick model. After superposing X-ray ligands Root Mean Square Deviation (RMSD) value is 0.029 Å. Docking score, glide gscore and glide emodel for compound **B24** were found as: −11.869, −11.869 and −96.645, respectively.

As stated in MAO inhibition assay, compound **B24** was found to be most active and selective derivative against MAO-A. Thus, to assess in silico its activity and observe the binding modes between **B24** and MAO-A, docking studies were carried out. The X-ray crystal structure of MAO-A (PDB ID: 2Z5X) [26] was obtained from the Protein Data Bank server (www.pdb.org). The docking poses of compound **B24** on MAO-A are presented in Figure 3.

By looking at the interactions described in the literature for harmine, it was seen that harmine is positioned in the active site of the enzyme MAO-A. It interacts with Tyr69, Asn181, Phe208, Val210, Gln215, Cys323, Ile325, Ile335, Leu337, Phe352, Tyr407, Tyr444 and flavin adenine dinucleotide (FAD) molecule. The amide group of the Gln215 side chain interacts tightly with harmine by a π–π interaction. Also, the pyridine ring is stabilized with Tyr407 and Tyr444 by forming a π-π interaction with phenyl rings [26,27]. When the docking pose of compound **B24** was analysed, both similar and additional interactions were observed. Compound **B24** closely binds to some amino acid residues lining the cavity, and is located very near the FAD cofactor. According to the docking pose (Figure 3), two π-π interactions are observed. 

The 4-chlorophenyl substructure is in an interaction with the phenyl of Phe208, whereas the benzylidene substructure establishes a π-π interaction with the phenyl of Phe352. Due to its hydrogen acceptor and donor atoms thiosemicarbazide moiety is essential for polar interactions. Two nitrogen atoms (N3 and N4) of this group have ability to form two different hydrogen bonds. The first hydrogen bond is observed between the amino nitrogen and the carbonyl of Asn181. The other one is seen between the imine nitrogen and hydroxyl of Tyr407. These interactions support the approach, which reveals that amino acid side chains, coating the cavity, are very favorable to interact with the amine moieties [28,29,30,31]. The last interaction in the structure is seen as a halogen bond. The chloro atom at the C4 position of the phenyl ring forms a halogen bond with Ala111. The appearance of this interaction explains why compound **B24** displays stronger binding to MAO-A.

When the chemical structures of the synthesized compounds are examined in general, it would be expected that compounds **B10** and **B11** should display similar activity to compound **B24**. However, their low inhibition rates may be due to the presence of the phenyl ring in place of a cyclohexyl. It is thought that the phenyl ring makes the structure more stable and leads a a rigid conformation. As a result, for compounds **B10** and **B11** it could be suggested that the presence of a phenyl on the N1 atom leads to less binding to the enzyme active site, which reduces their enzyme inhibition potency.

### 2.6. Theoretical Determination of Absorption, Distribution, Metabolism, and Excretion (ADME) Properties

The physicochemical properties of a compound are important for its suitability to be developed as an oral drug candidate. Absorption, distribution, metabolism, and excretion (ADME) studies were performed using the QikProp 4.8 software [32]. This software calculates any violations of Lipinski’s rule of five [33] and Jorgensen’s rule of three [34]. The results are exhibited in Table 2. Considering Lipkinski’s rule of five, compounds with a number of violations of not more than 1 show good bioavailability. Molecular weights (MW) of the all compounds are smaller than 500 Dalton. Their hydrogen bond donors (DHB), hydrogen bond acceptors (AHB) and polar surface area (PSA) values are within the range. Some of the compounds have an octanol/water partition coefficient (log P) value bigger than five. Compounds **B1**–**B24** are in general accordance with the rule by causing no more than one violation. In accordance with Jorgensen’s rule of three, all apparent Caco-2 cell permeability (PCaco) values are very high and number of likely primer metabolic reactions (PM) are within the limits. The aqueous solubility (log S) of the compounds are smaller than −5.7 and the only violation from this rule.

MAO inhibitors target to have effects on central nervous system (CNS), thus should able to pass the blood-brain barrier (BBB). The blood/brain partition coefficient (logBB), apparent Madin-Darby canine kidney (MDCK) cell permeability (PMDCK) are useful parameters to assign the penetration capacity of a compound from blood-brain barrier. The logBB values of all compounds are within recommended −3–+1.2 range. The PMDCK value of all compounds are higher than 500. The predicted CNS value of compound **B24** is 1, while this value is 0 for the rest of the compounds. Consequently, compounds **B1**–**B26** are determined to have good pharmacokinetic profiles and high BBB permeability, which enhances the biological importance of these compounds as potential CNS drug candidates.

## 3. Materials and Methods

### 3.1. General Information

All chemicals were purchased from Sigma-Aldrich Chemical Co. (St. Louis, MO, USA) and Merck KGaA (Darmstadt, Germany). All melting points (m.p.) were determined on an Electrothermal 9100 digital melting point apparatus (Electrothermal, Essex, UK) and are uncorrected. All reactions were monitored by thin-layer chromatography (TLC) using Silica Gel 60 F254 TLC plates (Merck KGaA). Spectroscopic data were recorded with the following instruments: IR: 8400S spectrophotometer (Shimadzu, Tokyo, Japan); ^1^H-NMR: DPX 300 NMR spectrometer (Bruker Bioscience, Billerica, MA, USA), ^13^C-NMR: Bruker DPX 75 NMR spectrometer in dimethyl sulfoxide (DMSO)-*d*_6_, using TMS as internal standard; HRMS: Shimadzu, Liquid Chromatography/Mass Spectrometer Ion-Trap and Time-of-Flight (LC/MS ITTOF) system.

### 3.2. Chemistry

#### 3.2.1. Synthesis of *N*-Phenyl/Cyclohexylhydrazinecarbothioamides **1**,**2**

Phenyl- or cyclohexylisothiocyanate (25 mmol) was dissolved in ethanol (200 mL). Hydrazine hydrate (50 mmol) was placed in an addition funnel and diluted with ethanol (20 mL). The diluted hydrazine hydrate was added to the mixture in an ice bath. After the completion of the addition, the resulting solid product was filtered and washed with cold ethanol. The products were recrystallized from ethanol.

#### 3.2.2. Synthesis of 4-Substituted Benzaldehyde Derivatives **3**–**16**

Appropriate seconder amines (5 mmol) or 4-substituted phenol/thiophenol derivatives (5 mmol) were refluxed with 4-fluorobenzaldehyde (5 mmol, 0.62 g) in dimethylformamide (10 mL) with the presence of potassium carbonate (6 mmol, 0.83 g). After TLC screening, the mixture was poured into ice water and filtered. The products were recrystallized from ethanol. 

#### 3.2.3. Synthesis of 2-(4-Substituted Benzylidene)-*N*-phenyl/cyclohexylhydrazine-1-carbothioamide Derivatives **B1**–**B26**

Equal amounts of *N*-phenyl/cyclohexylhydrazinecarbothioamides **1**,**2** (2 mmol) and the synthesized 4-substituted benzaldehyde (2 mmol) derivatives **3**–**16** were refluxed in ethanol (40 mL). After completion of the reaction, the product was filtered and recrystallized from ethanol.

*N-Phenyl-2-[4-(piperidin-1-yl)benzylidene]hydrazine-1-carbothioamide* (**B1**): Yield 68–70%, m.p. 182–184 °C. Fourier transform IR (FTIR) (Attenuated total reflection (ATR), cm^−1^): 3300 (N-H), 3134 (aromatic C-H), 2929 (aliphatic C-H), 1591–1444 (C=N and C=C), 1124 (C-N). ^1^H-NMR (300 MHz, DMSO-*d*_6_, ppm) δ 1.58 (6H, s, piperidine-H), 3.26 (4H, s, piperidine-H), 6.93 (2H, d, *J* = 8.9 Hz, disubstituted phenyl-H), 7.19 (1H, t, *J* = 7.4 Hz, phenyl-H), 7.36 (2H, t, *J* = 7.8 Hz, phenyl-H), 7.59 (2H, d, *J* = 7.9 Hz, phenyl-H), 7.69 (2H, d, *J* = 8.8 Hz, disubstituted phenyl-H), 8.05 (1H, s, N=CH), 9.95 (1H, s, NH), 11.60 (1H, s, NH). ^13^C-NMR (75 MHz, DMSO-*d*_6_, ppm) δ 24.37, 25.43, 48.85, 114.89, 123.39, 125.54, 126.03, 128.47, 129.43, 139.61, 144.10, 152.90, 175.55. HRMS (*m*/*z*): [M + H]^+^ calcd. for C_19_H_22_N_4_S: 339.1638; found 339.1626.

*N-phenyl-2-[4-(2-methylpiperidin-1-yl)benzylidene]hydrazine-1-carbothioamide* (**B2**)*:* Yield 68–70%, m.p. 178–180 °C. FTIR (ATR, cm^−1^): 3300 (N-H), 3132 (aromatic C-H), 2976 (aliphatic C-H), 1591–1444 (C=N and C=C), 1182 (C-N). ^1^H-NMR (300 MHz, DMSO-*d*_6_, ppm) δ 0.91 (3H, d, *J* = 6.6 Hz, CH_3_), 1.06–1.14 (1H, m, piperidine-H), 1.44–1.79 (4H, m, piperidine-H), 2.37–2.45 (1H, m, piperidine-H), 2.67–2.76 (1H, m, piperidine-H), 3.70–3.80 (2H, m, piperidine-H), 6.94 (2H, d, *J* = 8.9 Hz, disubstituted phenyl-H), 7.19 (1H, t, *J* = 7.3 Hz, phenyl-H), 7.36 (2H, t, *J* = 8.1 Hz, phenyl-H), 7.59 (2H, d, *J* = 7.5 Hz, phenyl-H), 7.69 (2H, d, *J* = 8.9 Hz, disubstituted phenyl-H), 8.04 (1H, s, N=CH), 9.94 (1H, s, NH), 11.59 (1H, s, NH). ^13^C-NMR (75 MHz, DMSO-*d*_6_, ppm) δ 19.65, 24.87, 30.54, 32.96, 48.25, 55.66, 114.82, 123.25, 125.54, 126.03, 128.47, 129.46, 139.62, 144.13, 152.68, 175.53. HRMS (*m*/*z*): [M + H]^+^ calcd. for C_20_H_24_N_4_S: 353.1794; found 353.1809.

*N-Phenyl-2-[4-(4-methylpiperidin-1-yl)benzylidene]hydrazine-1-carbothioamide* (**B3**)*:* Yield 68–70%, m.p. 156–159 °C. FTIR (ATR, cm^−1^): 3280 (N-H), 3134 (aromatic C-H), 2943 (aliphatic C-H), 1591–1442 (C=N and C=C), 1132 (C-N). ^1^H-NMR (300 MHz, DMSO-*d*_6_, ppm) δ 0.92 (3H, d, *J* = 6.5 Hz, CH_3_), 1.11–1.24 (2H, m, piperidine-H), 1.47–1.60 (1H, m, piperidine-H), 1.66–1.70 (2H, m, piperidine-H), 2.70–2.79 (2H, m, piperidine-H), 3.79–3.83 (2H, m, piperidine-H), 6.93 (2H, d, *J* = 9.0 Hz, disubstituted phenyl-H), 7.19 (1H, t, *J* = 7.3 Hz, phenyl-H), 7.36 (2H, t, *J* = 7.9 Hz, phenyl-H), 7.59 (2H, d, *J* = 7.5 Hz, phenyl-H), 7.69 (2H, d, *J* = 8.9 Hz, disubstituted phenyl-H), 8.04 (1H, s, N=CH), 9.94 (1H, s, NH), 11.60 (1H, s, NH). ^13^C-NMR (75 MHz, DMSO-*d*_6_, ppm) δ 22.22, 30.70, 33.68, 48.18, 114.90, 123.37, 125.54, 126.03, 128.47, 129.44, 139.61, 144.11, 152.68, 175.54. HRMS (*m*/*z*): [M + H]^+^ calcd. for C_20_H_24_N_4_S: 353.1794; found 353.1793.

*N-phenyl-2-[4-(4-phenylpiperazin-1-yl)benzylidene]hydrazine-1-carbothioamide* (**B4**)*:* Yield 68–70%, m.p. 214–217 °C. FTIR (ATR, cm^−1^): 3344 (N-H), 3118 (aromatic C-H), 2966 (aliphatic C-H), 1593–1444 (C=N and C=C), 1207 (C-N). ^1^H-NMR (300 MHz, DMSO-*d*_6_, ppm) δ 3.25–3.29 (4H, m, piperazine-H), 3.42 (4H, s, piperazine-H), 6.81 (1H, t, *J* = 7.2 Hz, phenyl-H), 7.01 (4H, m, disubstituted phenyl-H), 7.17–7.27 (3H, m, phenyl-H), 7.36 (2H, t, *J* = 8.1 Hz, phenyl-H), 7.58 (2H, d, *J* = 7.5 Hz, phenyl-H), 7.75 (2H, d, *J* = 8.9 Hz, disubstituted phenyl-H), 8.07 (1H, s, N=CH), 9.98 (1H, s, NH), 11.63 (1H, s, NH). ^13^C-NMR (75 MHz, DMSO-*d*_6_, ppm) δ 47.71, 48.62, 115.05, 116.14, 119.68, 124.45, 125.60, 126.14, 128.48, 129.41, 129.48, 139.61, 143.92, 151.29, 152.47, 175.70. HRMS (*m*/*z*): [M + H]^+^ calcd. for C_24_H_25_N_5_S: 416.1903; found 416.1892.

*N-phenyl-2-[4-[4-(4-methoxyphenyl)piperazin-1-yl]benzylidene]hydrazine-1-carbothioamide* (**B5**)*:* Yield 68–70%, m.p. 212–215 °C. FTIR (ATR, cm^−1^): 3334 (N-H), 3122 (aromatic C-H), 2974 (aliphatic C-H), 1591–1446 (C=N and C=C), 1269–1028 (C-N and C-O). ^1^H-NMR (300 MHz, DMSO-*d*_6_, ppm) δ 3.12–3.16 (4H, m, piperazine-H), 3.38 (4H, s, piperazine-H), 3.69 (3H, s, OCH_3_), 6.84 (2H, d, *J* = 9.1 Hz, methoxyphenyl-H), 6.95 (2H, d, *J* = 9.0 Hz, methoxyphenyl-H), 7.02 (2H, d, *J* = 8.9 Hz, disubstituted phenyl-H), 7.19 (1H, t, *J* = 7.5 Hz, phenyl-H), 7.36 (2H, t, *J* = 7.9 Hz, phenyl-H), 7.58 (2H, d, *J* = 7.5 Hz, phenyl-H), 7.75 (2H, d, *J* = 8.8 Hz, disubstituted phenyl-H), 8.07 (1H, s, N=CH), 9.98 (1H, s, NH), 11.64 (1H, s, NH). ^13^C-NMR (75 MHz, DMSO-*d*_6_, ppm) δ 47.86, 50.12, 55.66, 114.77, 115.05, 118.24, 124.43, 125.58, 126.14, 128.47, 129.40, 139.63, 143.90, 145.67, 152.51, 153.66, 175.70. HRMS (*m*/*z*): [M + H]^+^ calcd. for C_25_H_27_N_5_OS: 446.2009; found 446.1992.

*N-phenyl-2-[4-(4-methylphenoxy)benzylidene]hydrazine-1-carbothioamide* (**B6**)*:* Yield 68–70%, m.p. 175–178 °C. FTIR (ATR, cm^−1^): 3318 (N-H), 3113 (aromatic C-H), 2970 (aliphatic C-H), 1593–1449 (C=N and C=C), 1238–1157 (C-N and C-O). ^1^H-NMR (300 MHz, DMSO-*d*_6_, ppm) δ 2.31 (3H, s, CH_3_), 6.97–6.99 (4H, m, methylphenyl-H, disubstituted phenyl-H), 7.17–7.24 (3H, m, methylphenyl-H, phenyl-H), 7.37 (2H, t, *J* = 7.8 Hz, phenyl-H), 7.57 (2H, d, *J* = 7.8 Hz, phenyl-H), 7.90 (2H, d, *J* = 8.7 Hz, disubstituted phenyl-H), 8.13 (1H, s, N=CH), 10.08 (1H, s, NH), 11.78 (1H, s, NH). ^13^C-NMR (75 MHz, DMSO-*d*_6_, ppm) δ 20.77, 118.09, 119.92, 125.73, 126.30, 128.50, 129.19, 129.97, 131.02, 133.83, 139.54, 142.75, 153.80, 159.46, 176.24. HRMS (*m*/*z*): [M + H]^+^ calcd. for C_21_H_19_N_3_OS: 362.1322; found 362.1315.

*N-phenyl-2-[4-[(4-methylphenyl)thio]benzylidene]hydrazine-1-carbothioamide* (**B7**)*:* Yield 68–70%, m.p. 162–165 °C. FTIR (ATR, cm^−1^): 3420 (N-H), 3136 (aromatic C-H), 2978 (aliphatic C-H), 1591–1447 (C=N and C=C), 1263 (C-N). ^1^H-NMR (300 MHz, DMSO-*d*_6_, ppm) δ 2.32 (3H, s, CH_3_), 7.18 (2H, d, *J* = 8.4 Hz, disubstituted phenyl-H), 7.22–7.24 (3H, m, methylphenyl-H, phenyl-H), 7.33–7.39 (4H, m, methylphenyl-H, phenyl-H), 7.55 (2H, d, *J* = 7.7 Hz, phenyl-H), 7.84 (2H, d, *J* = 8.4 Hz, disubstituted phenyl-H), 8.09 (1H, s, N=CH), 10.08 (1H, s, NH), 11.82 (1H, s, NH). ^13^C-NMR (75 MHz, DMSO-*d*_6_, ppm) δ 21.19, 125.82, 126.30, 128.53, 128.61, 128.84, 129.32, 130.98, 132.50, 133.46, 138.87, 139.45, 139.75, 142.51, 176.33. HRMS (*m*/*z*): [M + H]^+^ calcd. for C_21_H_19_N_3_S_2_: 378.1093; found 378.1086.

*N-Phenyl-2-[4-(4-methoxyphenoxy)benzylidene]hydrazine-1-carbothioamide* (**B8**)*:* Yield 68–70%, m.p. 189–192 °C. FTIR (ATR, cm^−1^): 3306 (N-H), 3129 (aromatic C-H), 2968 (aliphatic C-H), 1595–1447 (C=N and C=C), 1254–1194 (C-N and C-O). ^1^H-NMR (300 MHz, DMSO-*d*_6_, ppm) δ 3.75 (3H, s, OCH_3_), 6.93 (2H, d, *J* = 8.8 Hz, disubstituted phenyl-H), 6.99 (2H, d, *J* = 9.2 Hz, methoxyphenyl-H), 7.06 (2H, d, *J* = 9.1 Hz, methoxyphenyl-H), 7.20 (1H, t, *J* = 7.4 Hz, phenyl-H), 7.36 (2H, t, *J* = 7.7 Hz, phenyl-H), 7.55 (2H, d, *J* = 7.3 Hz, phenyl-H), 7.81 (2H, d, *J* = 8.5 Hz, disubstituted phenyl-H), 8.09 (1H, s, N=CH), 10.08 (1H, s, NH), 11.82 (1H, s, NH). ^13^C-NMR (75 MHz, DMSO-*d*_6_, ppm) δ 55.90, 115.65, 117.40, 121.67, 125.76, 126.28, 128.51, 128.78, 129.94, 139.51, 142.86, 149.01, 156.47, 160.21, 176.20. HRMS (*m*/*z*): [M + H]^+^ calcd. for C_21_H_19_N_3_SO_2_: 378.1271; found 378.1263.

*N-Phenyl-2-[4-[(4-methoxyphenyl)thio]benzylidene]hydrazine-1-carbothioamide* (**B9**)*:* Yield 68–70%, m.p. 175–178 °C. FTIR (ATR, cm^−1^): 3289 (N-H), 3157 (aromatic C-H), 2974 (aliphatic C-H), 1591–1456 (C=N and C=C), 1287–1190 (C-N and C-O). ^1^H-NMR (300 MHz, DMSO-*d*_6_, ppm) δ 3.80 (3H, s, OCH_3_), 7.05 (2H, d, *J* = 8.9 Hz, methoxyphenyl-H), 7.09 (2H, d, *J* = 8.4 Hz, disubstituted phenyl-H), 7.20 (1H, t, *J* = 7.4 Hz, phenyl-H), 7.36 (2H, t, *J* = 7.8 Hz, phenyl-H), 7.47 (2H, d, *J* = 8.8 Hz, methoxyphenyl-H), 7.56 (2H, d, *J* = 7.7 Hz, phenyl-H), 7.88 (2H, d, *J* = 8.8 Hz, disubstituted phenyl-H), 8.11 (1H, s, N=CH), 10.05 (1H, s, NH), 11.75 (1H, s, NH). ^13^C-NMR (75 MHz, DMSO-*d*_6_, ppm) δ 55.82, 116.01, 122.14, 125.77, 126.28, 127.20, 128.50, 128.76, 131.94, 136.46, 139.49, 141.29, 142.53, 160.54, 176.28. HRMS (*m*/*z*): [M + H]^+^ calcd. for C_21_H_19_N_3_S_2_O: 394.1042; found 394.1034.

*N-Phenyl-2-[4-(4-chlorophenoxy)benzylidene]hydrazine-1-carbothioamide* (**B10**)*:* Yield 68–70%, m.p. 167–170 °C. FTIR (ATR, cm^−1^): 3318 (N-H), 3111 (aromatic C-H), 2966 (aliphatic C-H), 1589–1447 (C=N and C=C), 1236–1194 (C-N and C-O). ^1^H-NMR (300 MHz, DMSO-*d*_6_, ppm) δ 7.06 (2H, d, *J* = 8.8 Hz, disubstituted phenyl-H), 7.10 (2H, d, *J* = 8.9 Hz, chlorophenyl-H), 7.20 (1H, t, *J* = 7.4 Hz, phenyl-H), 7.37 (2H, t, *J* = 7.8 Hz, phenyl-H), 7.47 (2H, d, *J* = 8.9 Hz, chlorophenyl-H), 7.56 (2H, d, *J* = 7.4 Hz, phenyl-H), 7.95 (2H, d, *J* = 8.8 Hz, disubstituted phenyl-H), 8.14 (1H, s, N=CH), 10.10 (1H, s, NH), 11.81 (1H, s, NH). ^13^C-NMR (75 MHz, DMSO-*d*_6_, ppm) δ 118.94, 121.29, 125.77, 126.34, 128.25, 128.50, 129.49, 130.10, 130.50, 139.53, 142.57, 155.40, 158.39, 176.32. HRMS (*m*/*z*): [M + H]^+^ calcd. for C_20_H_16_N_3_SOCl: 382.0775; found 382.0764.

*N-Phenyl-2-[4-[(4-chlorophenyl)thio]benzylidene]hydrazine-1-carbothioamide* (**B11**)*:* Yield 68–70%, m.p. 175–177 °C. FTIR (ATR, cm^−1^): 3329 (N-H), 3127 (aromatic C-H), 2972 (aliphatic C-H), 1591–1447 (C=N and C=C), 1269–1203 (C-N and C-S). ^1^H-NMR (300 MHz, DMSO-*d*_6_, ppm) δ 7.20 (1H, t, *J* = 7.4 Hz, phenyl-H), 7.31 (2H, d, *J* = 8.4 Hz, disubstituted phenyl-H), 7.37–7.41 (4H, m, chlorophenyl-H, phenyl-H), 7.47 (2H, d, *J* = 8.7 Hz, chlorophenyl-H), 7.54 (2H, d, *J* = 7.4 Hz, phenyl-H), 7.90 (2H, d, *J* = 8.4 Hz, disubstituted phenyl-H), 8.12 (1H, s, N=CH), 10.12 (1H, s, NH), 11.85 (1H, s, NH). ^13^C-NMR (75 MHz, DMSO-*d*_6_, ppm) δ 125.86, 126.36, 128.54, 129.09, 130.16, 130.49, 133.19, 133.38, 133.51, 133.55, 137.31, 139.45, 142.32, 176.44. HRMS (*m*/*z*): [M + H]^+^ calcd. for C_20_H_16_N_3_S_2_Cl: 398.0547; found 398.0533.

*N-Phenyl-2-[4-(4-fluorophenoxy)benzylidene]hydrazine-1-carbothioamide* (**B12**)*:* Yield 68–70%, m.p. 135–136 °C. FTIR (ATR, cm^−1^): 3285 (N-H), 3126 (aromatic C-H), 2974 (aliphatic C-H), 1591–1495 (C=N and C=C), 1271–1192 (C-N and C-O). ^1^H-NMR (300 MHz, DMSO-*d*_6_, ppm) δ 7.00 (2H, d, *J* = 8.8 Hz, disubstituted phenyl-H), 7.11–7.14 (2H, m, fluorophenyl-H), 7.20 (1H, t, *J* = 7.3 Hz, phenyl-H), 7.24–7.20 (2H, m, fluorophenyl -H), 7.36 (2H, t, *J* = 7.8 Hz, phenyl-H), 7.54 (2H, d, *J* = 7.4 Hz, phenyl-H), 7.91 (2H, d, *J* = 8.8 Hz, disubstituted phenyl-H), 8.12 (1H, s, N=CH), 10.08 (1H, s, NH), 11.77 (1H, s, NH). ^13^C-NMR (75 MHz, DMSO-*d*_6_, ppm) δ 117.23 (d, *J* = 23.3 Hz), 118.16, 121.82 (d, *J* = 8.3 Hz), 125.79, 126.31, 128.52, 129.02 (d, *J* = 3.1 Hz), 129.47, 130.04, 139.50, 142.71, 159.29, 163.11 (d, *J* = 242.3 Hz), 176.27. HRMS (*m*/*z*): [M + H]^+^ calcd. for C_20_H_16_N_3_SOF: 366.1071; found 366.1061.

*N-Phenyl-2-[4-[(4-fluorophenyl)thio]benzylidene]hydrazine-1-carbothioamide* (**B13**)*:* Yield 68–70%, m.p. 168–169 °C. FTIR (ATR, cm^−1^): 3331 (N-H), 3127 (aromatic C-H), 2974 (aliphatic C-H), 1591–1447 (C=N and C=C), 1271–1153 (C-N and C-S). ^1^H-NMR (300 MHz, DMSO-*d*_6_, ppm) δ 7.20–7.23 (2H, m, disubstituted phenyl-H, phenyl-H), 7.27–7.39 (2H, m, fluorophenyl-H, phenyl-H), 7.50–7.57 (2H, m, fluorophenyl-H, phenyl-H), 7.87 (2H, d, *J* = 8.5 Hz, disubstituted phenyl-H), 8.10 (1H, s, N=CH), 10.10 (1H, s, NH), 11.85 (1H, s, NH). ^13^C-NMR (75 MHz, DMSO-*d*_6_, ppm) δ 117.43 (d, *J* = 21.8 Hz), 125.81, 126.34, 128.51, 128.81, 128.98 (d, *J* = 3.0 Hz), 132.89, 135.62 (d, *J* = 8.4 Hz), 139.03, 139.48, 142.36, 162.68 (d, *J* = 244.5 Hz), 176.39. HRMS (*m*/*z*): [M + H]^+^ calcd. for C_20_H_16_N_3_S_2_F: 382.0842; found 382.0831.

*N-Cyclohexyl-2-[4-(piperidin-1-yl)benzylidene]hydrazine-1-carbothioamide* (**B14**)*:* Yield 66–68%, m.p. 204–205 °C. FTIR (ATR, cm^−1^): 3310 (N-H), 3061 (aromatic C-H), 2987 (aliphatic C-H), 1600 (C=N), 1449 (C=C), 1269 (C-N). ^1^H-NMR (300 MHz, DMSO-*d*_6_, ppm) δ 1.08–1.88 (16H, m, cyclohexyl-H, piperidine-H), 3.25 (4H, br s, cyclohexyl-H, piperidine-H), 4.11–4.22 (1H, m, cyclohexyl-H, piperidine-H), 6.92 (2H, d, *J* = 8.9 Hz, disubstituted phenyl-H), 7.57 (2H, d, *J* = 8.8 Hz, disubstituted phenyl-H), 7.84 (1H, d, *J* = 8.6 Hz, N=CH), 7.94 (1H, s, NH), 11.19 (1H, s, NH). ^13^C-NMR (75 MHz, DMSO-*d*_6_, ppm) δ 24.38, 25.38, 25.44, 25.62, 32.39, 48.92, 52.85, 115.02, 123.61, 129.03, 143.27, 152.77, 178.58. HRMS (*m*/*z*): [M + H]^+^ calcd. for C_19_H_28_N_4_S: 345.2107; found 345.2091.

*N-Cyclohexyl-2-[4-(2-methylpiperidin-1-yl)benzylidene]hydrazine-1-carbothioamide* (**B15**)*:* Yield 68–70%, m.p. 212–213 °C. FTIR (ATR, cm^−1^): 3306 (N-H), 3051 (aromatic C-H), 2986 (aliphatic C-H), 1599 (C=N), 1452 (C=C), 1219 (C-N). ^1^H-NMR (300 MHz, DMSO-*d*_6_, ppm) δ 0.92 (3H, d, *J* = 6.5 Hz, CH_3_), 1.12–1.88 (16H, m, cyclohexyl-H, piperidine-H), 2.69–2.78 (2H, m, cyclohexyl-H, piperidine-H), 3.77–3.81 (2H, m, cyclohexyl-H, piperidine-H), 4.11–4.22 (1H, m, cyclohexyl-H, piperidine-H), 6.92 (2H, d, *J* = 8.9 Hz, disubstituted phenyl -H), 7.57 (2H, d, *J* = 8.9 Hz, disubstituted phenyl -H), 7.83 (1H, d, *J* = 8.6 Hz, N=CH), 7.94 (1H, s, NH), 11.18 (1H, s, NH). ^13^C-NMR (75 MHz, DMSO-*d*_6_, ppm) δ 22.22, 25.38, 25.62, 30.70, 32.39, 33.69, 48.25, 52.85, 115.02, 123.59, 129.04, 143.27, 152.55, 175.58. HRMS (*m*/*z*): [M + H]^+^ calcd. for C_20_H_30_N_4_S: 359.2264; found 359.2252.

*N-Cyclohexyl-2-[4-(4-methylpiperidin-1-yl)benzylidene]hydrazine-1-carbothioamide* (**B16**)*:* Yield 68–70%, m.p. 200–201 °C. FTIR (ATR, cm^−1^): 3310 (N-H), 3067 (aromatic C-H), 2976 (aliphatic C-H), 1599 (C=N), 1503 (C=C), 1218 (C-N). ^1^H-NMR (300 MHz, DMSO-*d*_6_, ppm) δ 0.91 (3H, d, *J* = 6.6 Hz, CH_3_), 1.02–1.89 (16H, m, cyclohexyl-H, piperidine-H), 2.40–2.44 (1H, m, cyclohexyl-H, piperidine-H), 2.65–2.74 (1H, m, cyclohexyl-H, piperidine-H), 3.68–3.76 (2H, m, cyclohexyl-H, piperidine-H), 4.16–4.18 (1H, m, cyclohexyl-H, piperidine-H), 6.92 (2H, d, *J* = 8.9 Hz, disubstituted phenyl -H), 7.56 (2H, d, *J* = 8.9 Hz, disubstituted phenyl-H), 7.83 (1H, d, *J* = 8.6 Hz, N=CH), 7.94 (1H, s, NH), 11.19 (1H, s, NH). ^13^C-NMR (75 MHz, DMSO-*d*_6_, ppm) δ 19.67, 24.87, 25.37, 25.62, 30.53, 32.39, 32.97, 48.33, 52.83, 55.75, 114.94, 123.46, 129.05, 143.23, 152.55, 175.57. HRMS (*m*/*z*): [M + H]^+^ calcd. for C_20_H_30_N_4_S: 359.2264; found 359.2262.

*N-Cyclohexyl-2-[4-(4-phenylpiperazin-1-yl)benzylidene]hydrazine-1-carbothioamide* (**B17**)*:* Yield 68–70%, m.p. 227–228 °C. FTIR (ATR, cm^−1^): 3300 (N-H), 3061 (aromatic C-H), 2972 (aliphatic C-H), 1597 (C=N),1493 (C=C), 1229 (C-N). ^1^H-NMR (300 MHz, DMSO-*d*_6_, ppm) δ 1.03–1.89 (10H, m, cyclohexyl-H), 3.26–3.29 (4H, m, piperazine-H), 3.38–3.41 (4H, m, piperazine-H), 4.12–4.23 (1H, m, cyclohexyl-H), 6.81 (1H, t, *J* = 7.3 Hz, phenyl-H), 6.98–7.03 (4H, m, phenyl-H, disubstituted phenyl-H), 7.24 (2H, t, *J* = 8.0 Hz, phenyl-H), 7.64 (2H, d, *J* = 8.9 Hz, disubstituted phenyl-H), 7.88 (1H, d, *J* = 8.6 Hz, N=CH), 7.91 (1H, s, NH), 11.23 (1H, s, NH). ^13^C-NMR (75 MHz, DMSO-*d*_6_, ppm) δ 25.40, 25.63, 32.38, 47.79, 48.62, 52.92, 115.16, 116.14, 119.66, 124.64, 129.02, 129.46, 143.10, 151.31, 152.33, 175.66. HRMS (*m*/*z*): [M + H]^+^ calcd. for C_24_H_31_N_5_S: 422.2373; found 422.2357.

*N-Cyclohexyl-2-[4-[4-(4-methoxyphenyl)piperazin-1-yl]benzylidene]hydrazine-1-carbothioamide* (**B18**): Yield 68–70%, m.p. 238–239 °C. FTIR (ATR, cm^−1^): 3333 (N-H), 3052 (aromatic C-H), 2974 (aliphatic C-H), 1599 (C=N), 1489 (C=C), 1227–1107 (C-N and C-O). ^1^H-NMR (300 MHz, DMSO-*d*_6_, ppm) δ 1.12–1.89 (10H, m, cyclohexyl-H), 3.14 (4H, br s, piperazine-H), 3.37 (4H, br s, piperazine-H), 3.69 (3H, s, OCH_3_), 4.17–4.19 (1H, m, cyclohexyl-H), 6.84 (2H, d, *J* = 8.0 Hz, methoxyphenyl-H), 6.95 (2H, d, *J* = 8.1 Hz, methoxyphenyl-H), 7.01 (2H, d, *J* = 7.9 Hz, disubstituted phenyl-H), 7.63 (2H, d, *J* = 7.7 Hz, phenyl-H), 7.88 (1H, d, *J* = 8.1 Hz, N=CH), 7.97 (1H, s, NH), 11.23 (1H, s, NH). ^13^C-NMR (75 MHz, DMSO-*d*_6_, ppm) δ 25.40, 25.63, 32.38, 47.93, 50.12, 52.91, 55.66, 114.76, 115.15, 118.22, 124.61, 129.01, 143.10, 145.67, 152.37, 153.66, 175.66. HRMS (*m*/*z*): [M + H]^+^ calcd. for C_25_H_33_N_5_OS: 452.2479; found 452.2460.

*N-Cyclohexyl-2-[4-(4-methylphenoxy)benzylidene]hydrazine-1-carbothioamide* (**B19**)*:* Yield 68–70%, m.p. 169–170 °C. FTIR (ATR, cm^−1^): 3312 (N-H), 3057 (aromatic C-H), 2988 (aliphatic C-H), 1595 (C=N), 1497 (C=C), 1269–1076 (C-N and C-O). ^1^H-NMR (300 MHz, DMSO-*d*_6_, ppm) δ 1.08–1.94 (10H, m, cyclohexyl-H), 2.30 (3H, s, CH_3_), 4.12–4.24 (1H, m, cyclohexyl-H), 6.96–6.98 (4H, m, disubstituted phenyl-H, methylphenyl-H), 7.23 (2H, d, *J* = 8.4 Hz, methylphenyl-H), 7.78 (2H, d, *J* = 8.7 Hz, disubstituted phenyl-H), 7.97 (1H, d, *J* = 8.6 Hz, N=CH), 8.03 (1H, s, NH), 11.36 (1H, s, NH). ^13^C-NMR (75 MHz, DMSO-*d*_6_, ppm) δ 20.77 (CH_3_), 25.40, 25.62, 32.30, 53.05, 118.16, 119.85, 129.29, 129.62, 131.00, 133.78, 142.00, 153.84, 159.25, 176.01. HRMS (*m*/*z*): [M + H]^+^ calcd. for C_21_H_25_N_3_OS: 368.1791; found 368.1784.

*N-Cyclohexyl-2-[4-[(4-methylphenyl)thio]benzylidene]hydrazine-1-carbothioamide* (**B20**)*:* Yield 68–70%, m.p. 176–177 °C. FTIR (ATR, cm^−1^): 3306 (N-H), 3067 (aromatic C-H), 2986 (aliphatic C-H), 1593 (C=N), 1491 (C=C), 1209 (C-N). ^1^H-NMR (300 MHz, DMSO-*d*_6_, ppm) δ 1.03–1.87 (10H, m, cyclohexyl-H), 2.33 (3H, s, CH_3_), 4.11–4.24 (1H, m, cyclohexyl-H), 7.17 (2H, d, *J* = 8.4 Hz, disubstituted phenyl-H), 7.26 (2H, d, *J* = 8.1 Hz, methylphenyl-H), 7.35 (2H, d, *J* = 8.1 Hz, methylphenyl-H), 7.72 (2H, d, *J* = 8.4 Hz, disubstituted phenyl-H), 7.99 (1H, d, *J* = 8.6 Hz, N=CH), 7.99 (1H, s, NH), 11.42 (1H, s, NH). ^13^C-NMR (75 MHz, DMSO-*d*_6_, ppm) δ 21.18, 25.40, 25.62, 32.26, 53.10, 128.54, 128.68, 129.40, 130.96, 132.62, 133.42, 138.82, 139.41, 141.69, 176.07. HRMS (*m*/*z*): [M + H]^+^ calcd. for C_21_H_25_N_3_S_2_: 384.1563; found 384.1545.

*N-Cyclohexyl-2-[4-(4-methoxyphenoxy)benzylidene]hydrazine-1-carbothioamide* (**B21**)*:* Yield 68–70%, m.p. 159–160 °C. FTIR (ATR, cm^−1^): 3312 (N-H), 3051 (aromatic C-H), 2986 (aliphatic C-H), 1535 (C=N), 1495 (C=C), 1229–1076 (C-N and C-O). ^1^H-NMR (300 MHz, DMSO-*d*_6_, ppm) δ 1.03–1.88 (10H, m, cyclohexyl-H), 3.76 (3H, s, OCH_3_), 4.13–4.23 (1H, m, cyclohexyl-H), 6.93 (2H, d, *J* = 8.7 Hz, disubstituted phenyl-H), 6.98 (2H, d, *J* = 9.0 Hz, methoxyphenyl-H), 7.05 (2H, d, *J* = 9.1 Hz, methoxyphenyl-H), 7.76 (2H, d, *J* = 8.7 Hz, disubstituted phenyl-H), 7.96 (1H, d, *J* = 8.6 Hz, N=CH), 8.02 (1H, s, NH), 11.36 (1H, s, NH). ^13^C-NMR (75 MHz, DMSO-*d*_6_, ppm) δ 25.40, 25.62, 32.31, 53.03, 55.90, 115.64, 117.44, 121.64, 128.90, 129.58, 142.05, 149.05, 156.46, 160.01, 176.00. HRMS (*m*/*z*): [M + H]^+^ calcd. for C_21_H_25_N_3_O_2_S: 384.1740; found 384.1736.

*N-Cyclohexyl-2-[4-[(4-methoxyphenyl)thio]benzylidene]hydrazine-1-carbothioamide* (**B22**)*:* Yield 68–70%, m.p. 146–147 °C. FTIR (ATR, cm^−1^): 3308 (N-H), 3049 (aromatic C-H), 2974 (aliphatic C-H), 1591 (C=N), 1493 (C=C), 1242 (C-N). ^1^H-NMR (300 MHz, DMSO-*d*_6_, ppm) δ 1.09–1.87 (10H, m, cyclohexyl-H), 3.80 (3H, s, OCH_3_), 4.11–4.30 (1H, m, cyclohexyl-H), 7.46 (2H, d, *J* = 8.9 Hz, methoxyphenyl-H), 7.08 (2H, d, *J* = 8.3 Hz, disubstituted phenyl-H), 7.46 (2H, d, *J* = 8.7 Hz, methoxyphenyl-H), 7.69 (2H, d, *J* = 8.4 Hz, disubstituted phenyl-H), 7.95–7.98 (2H, m, N=CH, NH), 11.41 (1H, s, NH). ^13^C-NMR (75 MHz, DMSO-*d*_6_, ppm) δ 25.40, 25.62, 32.27, 53.07, 55.84, 116.00, 122.19, 127.27, 128.45, 130.96, 132.04, 136.42, 141.00, 141.77, 160.53, 176.04. HRMS (*m*/*z*): [M + H]^+^ calcd. for C_21_H_25_N_3_OS_2_: 400.1512; found 400.1506.

*N-Cyclohexyl-2-[4-(4-chlorophenoxy)benzylidene]hydrazine-1-carbothioamide* (**B23**)*:* Yield 68–70%, m.p. 176–177 °C. FTIR (ATR, cm^−1^): 3308 (N-H), 3063 (aromatic C-H), 2988 (aliphatic C-H), 1589 (C=N), 1483 (C=C), 1234–1076 (C-N and C-O). ^1^H-NMR (300 MHz, DMSO-*d*_6_, ppm) δ 1.06–1.88 (10H, m, cyclohexyl-H), 4.14–4.24 (1H, m, cyclohexyl-H), 7.05 (2H, d, *J* = 8.7 Hz, disubstituted phenyl-H), 7.09 (2H, d, *J* = 8.9 Hz, chlorophenyl-H), 7.46 (2H, d, *J* = 8.9 Hz, chlorophenyl-H), 7.82 (2H, d, *J* = 8.8 Hz, disubstituted phenyl-H), 8.00 (1H, d, *J* = 8.6 Hz, N=CH), 8.04 (1H, s, NH), 11.39 (1H, s, NH). ^13^C-NMR (75 MHz, DMSO-*d*_6_, ppm) δ 25.25, 25.41, 25.62, 32.29, 53.08, 119.01, 121.23, 128.20, 129.75, 130.14, 130.49, 141.82, 155.43, 158.18, 176.06. HRMS (*m*/*z*): [M + H]^+^ calcd. for C_20_H_22_ClN_3_OS: 388.1245; found 388.1228.

*N-Cyclohexyl-2-[4-[(4-chlorophenyl)thio]benzylidene]hydrazine-1-carbothioamide* (**B24**)*:* Yield 68–70%, m.p. 200–201 °C. FTIR (ATR, cm^−1^): 3306 (N-H), 3051 (aromatic C-H), 2980 (aliphatic C-H), 1593 (C=N), 1474 (C=C), 1209 (C-N). ^1^H-NMR (300 MHz, DMSO-*d*_6_, ppm) δ 1.10–1.88 (10H, m, cyclohexyl-H), 4.12–4.25 (1H, m, cyclohexyl-H), 7.31 (2H, d, *J* = 8.4 Hz, disubstituted phenyl-H), 7.38 (2H, d, *J* = 8.7 Hz, chlorophenyl-H), 7.47 (2H, d, *J* = 8.7 Hz, chlorophenyl-H), 7.79 (2H, d, *J* = 8.4 Hz, disubstituted phenyl-H), 8.01–8.05 (2H, m, N=CH, NH), 11.45 (1H, s, NH). ^13^C-NMR (75 MHz, DMSO-*d*_6_, ppm) δ 25.41, 25.62, 32.25, 53.14, 128.79, 130.15, 130.61, 133.12, 133.40, 133.49, 133.69, 136.95, 141.51, 176.13. HRMS (*m*/*z*): [M + H]^+^ calcd. for C_20_H_22_ClN_3_S_2_: 404.1016; found 404.1001.

*N-Cyclohexyl-2-[4-(4-fluorophenoxy)benzylidene]hydrazine-1-carbothioamide* (**B25**)*:* Yield 68–70%, m.p. 175–176 °C. FTIR (ATR, cm^−1^): 3314 (N-H), 3063 (aromatic C-H), 2976 (aliphatic C-H), 1593 (C=N), 1476 (C=C), 1248–1088 (C-N and C-O). ^1^H-NMR (300 MHz, DMSO-*d*_6_, ppm) δ 1.05–1.91 (10H, m, cyclohexyl-H), 4.12–4.37 (1H, m, cyclohexyl-H), 6.99 (2H, d, *J* = 8.8 Hz, disubstituted phenyl-H), 7.11–7.15 (2H, m, fluorophenyl-H), 7.23–7.29 (2H, m, fluorophenyl-H), 7.80 (2H, d, *J* = 8.8 Hz, disubstituted phenyl-H), 7.98 (1H, d, *J* = 8.6 Hz, N=CH), 8.03 (1H, s, NH), 11.38 (1H, s, NH). ^13^C-NMR (75 MHz, DMSO-*d*_6_, ppm) δ 25.40, 25.62, 32.30, 53.06, 112.14, 117.21 (d, *J* = 23.3 Hz), 118.22, 121.75 (d, *J* = 8.3 Hz), 129.64 (d, *J* = 6.0 Hz), 141.90, 152.26, 159.01 (d, *J* = 238.5 Hz), 159.08, 176.03. HRMS (*m*/*z*): [M + H]^+^ calcd. for C_20_H_22_FN_3_OS: 372.1540; found 372.1534.

*N-Cyclohexyl-2-[4-[(4-fluorophenyl)thio]benzylidene]hydrazine-1-carbothioamide* (**B26**)*:* Yield 68–70%, m.p. 173–174 °C. FTIR (ATR, cm^−1^): 3308 (N-H), 3067 (aromatic C-H), 2987 (aliphatic C-H), 1591 (C=N), 1487 (C=C), 1225 (C-N). ^1^H-NMR (300 MHz, DMSO-*d*_6_, ppm) δ 1.05–1.87 (10H, m, cyclohexyl-H), 4.12–4.24 (1H, m, cyclohexyl-H), 7.21 (2H, d, *J* = 8.3 Hz, disubstituted phenyl-H), 7.27–7.33 (2H, m, fluorophenyl-H), 7.48–7.53 (2H, m, fluorophenyl-H), 7.75 (2H, d, *J* = 8.4 Hz, disubstituted phenyl-H), 7.99–8.02 (2H, m, N=CH and NH),11.43 (1H, s, NH). ^13^C-NMR (75 MHz, DMSO-*d*_6_, ppm) δ 25.41, 25.62, 32.26, 53.12, 117.42 (d, *J* = 21.8 Hz), 128.65, 128.86, 129.08, 132.99, 135.56 (d, *J* = 8.3 Hz), 138.72, 141.61, 162.66 (d, *J* = 244.5 Hz), 176.09. HRMS (*m*/*z*): [M + H]^+^ calcd. for C_20_H_22_FN_3_S_2_: 388.1312; found 388.1300.

### 3.3. MAO Inhibition Assay

MAO inhibition assay was applied in concert with our recent study [35]. Ampliflu Red (10-Acetyl-3,7-dihydroxyphenoxazine), peroxidase from horseradish, MAO-A, MAO-B, H_2_O_2_, tyramine hydrochloride, moclobemide and selegiline were purchased from Sigma-Aldrich Chemical Co. (St. Louis, MO, USA) and retained under the suggested conditions by supplier. All pipetting processes were performed using a BioTek Precision XS robotic system (BioTek Instruments, Winooski, VT, USA). Measurements were carried out by a BioTek-Synergy H1 microplate reader (USA) based on the fluorescence generated (excitation, 535 nm, emission, 587 nm) over a 30 min period, in which the fluorescence increased linearly.

In the enzymatic assay, three different daily prepared solutions, consisting of inhibitor solutions, enzyme solutions and working solution, were used. Synthesized compounds and reference agents were prepared in 2% DMSO in 10^−3^–10^−9^ M concentrations (10 mL for each concentration) in order to gain inhibitor solution. Enzyme solutions were prepared using recombinant MAO-A (0.5 U/mL) and recombinant MAO-B (0.64 U/mL). These enzymes were dissolved in the phosphate buffer and final volumes were adjusted to 10 mL. Working solution consist of three main components: Horseradish peroxidase (200 U/mL, 100 µL), Ampliflu Red (20 mM, 200 µL) and tyramine (100 mM, 200 µL). These components were dissolved in the phosphate buffer and final volume was adjusted to 10 mL.

The solutions of inhibitor (20 µL/well) and MAO-A (100 µL/well) or MAO-B (100 µL/well) were added to the flat black bottom 96-well micro test plate, and incubated at 37 °C for 30 min. After this incubation period, working solution (100 µL/well) were added into each well to start the reaction. The mixture was incubated at 37 °C for 30 min and the fluorescence (Excitation/emission = 535/587 nm) was measured at 5 min intervals. For control experiments, 2% DMSO (20 µL) were used simultaneously by replacing the inhibitor solution. In addition, the possible capacity of the inhibitors to modify the fluorescence generated in the reaction mixture due to non-enzymatic inhibition was determined by mixing inhibitor and working solutions.

The specific fluorescence emission that used to obtain the final results was calculated after subtraction of the background activity, which was determined from vials containing all components except the MAO isoforms, which were replaced by phosphate buffer (100 μL/well). Blank, control and all concentrations of inhibitors were analyzed in quadruplicate and inhibition percent was calculated by using following equation:

$$\%\text{Inhibition}=\frac{\left(FCt_{2}-FCt_{1}\right)-\left(FIt_{2}-FIt_{1}\right)}{FCt_{2}-FCt_{1}}\times 100$$

*FCt*_2_: Fluorescence of a control well measured at *t*_2_ time, *FCt*_1_: Fluorescence of a control well measured at *t*_2_ time, *FIt*_2_: Fluorescence of an inhibitor well measured at *t*_2_ time, *FIt*_1_: Fluorescence of an inhibitor well measured at *t*_1_ time, The IC_50_ values were calculated from a dose-response curve obtained by plotting the percentage inhibition versus the log concentration with the use of GraphPad PRISM software (version 5.0, GraphPad Software Inc., La Jolla, CA, USA). The results were displayed as mean ± standard deviation (SD).

### 3.4. Enzyme Kinetic Studies

The similar protocol of MAO inhibition assay was followed by using the same materials. The most active compound **B24** was tested at the concentrations of IC_50_/2, IC_50_, and 2 × IC_50_. The solutions of compound **B24** (20 μL/well) and MAO-B enzyme were added to the flat black bottom 96-well micro test plate, and incubated at 37 °C for 30 min. Once incubation was completed, the working solution, including various concentrations (20, 10, 5, 2.5, 1.25 and 0.625 μM) of substrate (100 μL/well) was added. The increase of the fluorescence (Excitation/emission = 535/587 nm) was recorded for 30 min. A parallel experiment was carried out without inhibitor. All experiments were performed in quadruplicate. The experimental data were analyzed as Lineweaver-Burk plots using Microsoft Office Excel 2013. The K_m_/V_max_ (slope) values of the Lineweaver-Burk plots were replotted versus the inhibitor concentration to determine K_i_ values from the x-axis intercept as -K_i_.

### 3.5. Cytotoxicity Test

The cytotoxic potency of the most active compound **B24** was measured by MTT method [25] using the NIH/3T3 mouse embryonic fibroblast cell line (ATCC CRL-1658, London, UK) which is recommended for cytotoxicity screening by ISO (10993-5, 2009) [36]. NIH/3T3 cells were incubated according to the supplier’s recommendations. NIH/3T3 cells were seeded at 1 × 10^4^ cells into each well of 96-well plates. After this process, cells were treated with the compounds at concentrations ranging from 1000 µM to 0.316 µM. The IC_50_ value was determined by plotting a dose-response curve of inhibition % versus compound concentrations applied [37].

### 3.6. Molecular Docking Studies

A structure based in silico procedure was applied to discover the interaction modes between compound **B24** and MAO-A enzyme active site. The crystal structures of MAO-A (PDB ID: 2Z5X) [26], which was crystallized with the reversible inhibitor harmine, was retrieved from the Protein Data Bank server (www.pdb.org).

The structures of ligands were built using the Schrödinger Maestro [38] interface and then were submitted to the Protein Preparation Wizard protocol of the Schrödinger Suite 2016 Update 2 [39]. The ligands were prepared by the LigPrep 3.8 [40] to assign the protonation states at pH 7.4 ± 1.0 and the atom types, correctly. Bond orders were assigned, and hydrogen atoms were added to the structures. The grid generation was formed using Glide 7.1 [41]. The grid box with dimensions of 20 Å × 20 Å × 20 Å was centered in the vicinity of the flavin (FAD) N5 atom on the catalytic site of the protein to cover all binding sites and neighboring residues [1,42]. Flexible docking runs were performed with single precision docking mode (SP). 

### 3.7. Theoretical Determination of ADME Properties

Physicochemical properties and BBB permeability of the compounds were predicted via QikProp 4.8 software [32].

## 4. Conclusions

In this paper, we focused on the synthesis and biological evaluation of some novel thiosemicarbazones as MAO inhibitors. According to an activity assay, compound **B24** was determined as the most active molecule amongst all prepared compounds. Enzyme kinetics and docking studies clarified the type of interaction and binding modes between the enzyme and compounds. Compound **B24** was found to be a reversible and competitive inhibitor of MAO-A, that additionally shows molecular interactions with the active site of MAO-A in a modelling study. Cytotoxicity and ADME studies further indicated the potential biological importance of this compound. Based on these results, new molecules derived from compound **B24**, targeting MAO isoforms selectively, could possibly afford more promising drugs for the treatment of neurological diseases.

## Figures and Tables

**Table:** 

Compound	A	X	Secondary Amine	R	Compound	A	X	Secondary Amine	R
**B1**	phenyl	-	piperidine	-	**B14**	cyclohexyl	-	piperidine	-
**B2**	phenyl	-	2-metilpiperidine	-	**B15**	cyclohexyl	-	2-metilpiperidine	-
**B3**	phenyl	-	4-metilpiperidine	-	**B16**	cyclohexyl	-	4-metilpiperidine	-
**B4**	phenyl	-	4-phenylpiperazine	-	**B17**	cyclohexyl	-	4-phenylpiperazine	-
**B5**	phenyl	-	(4-methoxyphenyl)piperazine	-	**B18**	cyclohexyl	-	(4-methoxyphenyl)piperazine	-
**B6**	phenyl	O	-	CH_3_	**B19**	cyclohexyl	O	-	CH_3_
**B7**	phenyl	S	-	CH_3_	**B20**	cyclohexyl	S	-	CH_3_
**B8**	phenyl	O	-	OCH_3_	**B21**	cyclohexyl	O	-	OCH_3_
**B9**	phenyl	S	-	OCH_3_	**B22**	cyclohexyl	S	-	OCH_3_
**B10**	phenyl	O	-	Cl	**B23**	cyclohexyl	O	-	Cl
**B11**	phenyl	S	-	Cl	**B24**	cyclohexyl	S	-	>Cl
**B12**	phenyl	O	-	F	**B25**	cyclohexyl	O	-	F
**B13**	phenyl	S	-	F	**B26**	cyclohexyl	S	-	F

**Table 1:** Initial screening results to determine inhibition potency of compounds **B1**–**B26**, moclobemide and selegiline against monoamine oxidase A (MAO-A) and monoamine oxidase B (MAO-B) at concentrations of 10^−3^ M and 10^−4^ M.

Compound	MAO-A Inhibition %		MAO-B Inhibition %	
	10^−^^3^ M	10^−^^4^ M	10^−^^3^ M	10^−^^4^ M
**B1**	21.08 ± 0.34	13.77 ± 0.29	15.80 ± 0.30	10.21 ± 0.14
**B2**	30.68 ± 0.85	16.28 ± 0.97	27.08 ± 0.46	18.83 ± 0.37
**B3**	20.77 ± 0.55	15.51 ± 0.50	17.85 ± 0.28	11.41 ± 0.33
**B4**	30.85 ± 1.07	26.77 ± 0.79	20.32 ± 0.67	16.10 ± 0.62
**B5**	32.61 ± 1.03	16.63 ± 0.51	29.88 ± 0.88	14.43 ± 0.41
**B6**	26.98 ± 0.77	11.79 ± 0.28	22.58 ± 0.62	16.77 ± 0.44
**B7**	30.10 ± 1.20	24.08 ± 0.81	23.45 ± 0.16	18.79 ± 0.27
**B8**	34.83 ± 0.79	19.76 ± 0.71	25.17 ± 0.82	20.25 ± 0.80
**B9**	24.56 ± 0.89	18.80 ± 0.62	18.62 ± 0.40	14.11 ± 0.36
**B10**	40.79 ± 1.23	32.88 ± 0.77	20.43 ± 0.58	16.02 ± 0.69
**B11**	35.90 ± 0.22	30.01 ± 0.45	31.34 ± 0.74	27.85 ± 0.61
**B12**	32.06 ± 0.68	26.33 ± 0.57	29.66 ± 1.02	16.88 ± 0.47
**B13**	36.65 ± 1.13	27.19 ± 0.84	27.49 ± 0.66	20.66 ± 0.73
**B14**	41.08 ± 1.26	27.09 ± 0.67	23.47 ± 0.48	18.65 ± 0.33
**B15**	39.48 ± 1.05	20.06 ± 0.97	22.17 ± 0.88	14.20 ± 0.41
**B16**	37.96 ± 1.00	29.88 ± 0.98	24.63 ± 0.48	21.74 ± 0.36
**B17**	48.83 ± 1.02	39.77 ± 1.26	30.75 ± 0.78	17.49 ± 0.77
**B18**	39.40 ± 0.62	31.28 ± 0.57	26.79 ± 0.49	22.62 ± 0.40
**B19**	53.56 ± 1.08	40.57 ± 1.12	32.06 ± 0.81	27.05 ± 0.69
**B20**	41.33 ± 0.39	35.66 ± 0.27	28.20 ± 0.66	21.63 ± 0.50
**B21**	58.52 ± 1.36	35.75 ± 0.91	37.03 ± 0.40	30.49 ± 0.38
**B22**	50.88 ± 1.09	38.29 ± 0.97	27.88 ± 0.45	20.22 ± 0.41
**B23**	64.85 ± 1.04	46.10 ± 0.77	24.55 ± 0.68	19.62 ± 0.44
**B24**	83.31 ± 1.08	70.07 ± 1.12	14.43 ± 0.26	11.79 ± 0.30
**B25**	57.14 ± 1.17	32.38 ± 0.68	30.40 ± 0.60	24.61 ± 0.43
**B26**	51.33 ± 0.38	36.09 ± 0.22	27.99 ± 0.19	19.89 ± 0.11
**Moclobemide**	91.42 ± 4.60	77.86 ± 3.71	-	-
**Selegiline**	-	-	97.69 ± 4.16	94.42 ± 3.89

**Table 2:** Calculated Absorption, Distribution, Metabolism, and Excretion (ADME) parameters of compounds **B1**–**B26**.

Compound	MW	DHB	AHB	PSA	logP	logS	PCaco	PM	PMDCK	logBB	CNS	VRF	VRT
**B1**	338.470	2	5	49.573	4.334	−6.156	2663.609	2	2461.245	−0.222	0	0	1
**B2**	352.496	2	5	49.147	4.574	−6.362	2679.674	2	2477.294	−0.212	0	0	1
**B3**	352.496	2	5	49.575	4.660	−6.598	2663.218	2	2460.854	−0.231	0	0	1
**B4**	415.555	2	6	55.441	5.471	−7.686	2584.097	2	2381.928	−0.281	0	1	1
**B5**	445.581	2	6.75	63.922	5.519	−7.822	2584.100	3	2381.931	−0.352	0	1	1
**B6**	361.461	2	4.5	52.430	4.937	−6.253	2720.949	3	2518.564	−0.333	0	0	1
**B7**	377.521	2	4	44.334	5.540	−7.067	2720.949	3	4269.676	−0.249	0	1	1
**B8**	377.460	2	5.25	60.912	4.696	−5.858	2720.951	3	2518.566	−0.388	0	0	1
**B9**	393.521	2	4.75	52.815	5.296	−6.666	2720.951	3	4269.729	−0.304	0	1	1
**B10**	381.879	2	4.5	52.431	5.129	−6.434	2720.949	2	6213.625	−0.154	0	1	1
**B11**	397.939	2	4	44.334	5.730	−7.247	2720.949	2	10,000	−0.070	0	1	1
**B12**	365.424	2	4.5	52.431	4.873	−6.063	2720.949	2	4554.137	−0.207	0	0	1
**B13**	381.485	2	4	44.334	5.473	-6.872	2720.949	2	7721.149	−0.123	0	1	1
**B14**	344.517	2	5	47.948	4.598	−6.731	3310.935	0	3266.539	−0.115	0	0	1
**B15**	358.544	2	5	47.521	4.838	−6.938	3331.393	0	3288.360	−0.106	0	0	1
**B16**	358.544	2	5	47.989	4.898	−7.145	3204.223	0	3153.046	−0.138	0	0	1
**B17**	421.602	2	6	54.099	5.652	−8.096	2974.126	0	2908.896	−0.199	0	1	1
**B18**	451.629	2	6.75	62.782	5.827	−8.356	3504.952	1	3665.702	−0.174	0	1	1
**B19**	367.508	2	4.5	50.803	5.204	−6.835	3382.919	1	3343.532	−0.226	0	1	1
**B20**	383.569	2	4	42.707	5.810	−7.656	3382.919	1	5668.228	−0.139	0	1	1
**B21**	383.507	2	5.25	59.285	4.959	−6.433	3382.922	1	3343.534	−0.282	0	0	1
**B22**	399.568	2	4.75	51.188	5.562	−7.249	3382.922	1	5668.299	−0.194	0	1	1
**B23**	387.926	2	4.5	50.804	5.395	−7.015	3382.919	0	8248.927	−0.048	0	1	1
**B24**	403.987	2	4	42.707	6	−7.835	3382.919	0	10,000	-0.040	1	1	1
**B25**	371.472	2	4.5	50.804	5.138	−6.642	3382.919	0	6045.866	−0.102	0	1	1
**B26**	387.532	2	4	42.707	5.741	−7.459	3382.919	0	10,000	−0.015	0	1	1

MW: Molecular weight; DHB: Estimated number of hydrogen bond donors; AHB: Estimated number of hydrogen bond acceptors; PSA: Van der Waals surface area of polar nitrogen and oxygen atoms and carbonyl carbon atoms; logP: Predicted octanol/water partition coefficient; logS: Predicted aqueous solubility; PCaco: Predicted apparent Caco-2 cell permeability; PM: Number of likely metabolic reactions; PMDCK: Predicted apparent Madin-Darby canine kidney (MDCK) cell permeability; logBB: Predicted brain/blood partition coefficient; CNS: Predicted central nervous system activity on a −2 (inactive) to +2 (active) scale (recommended value: −2 (inactive), +2 (active));VRF: Number of violations of Lipinski’s rule of five. The rules are: MW < 500, logP < 5, DHB ≤ 5, AHB ≤ 10, Positive PSA value; VRT: Number of violations of Jorgensen’s rule of three. The three rules are: logS > −5.7, PCaco > 22 nm/s, PM < 7.

## Supplementary Materials

The Supplementary Materials are available online, Figure S1: Compound **B24** IR spectrum, Figure S2: Compound **B24** mass spectrum, Figure S3: Compound **B24** ^1^H-NMR spectrum, Figure S4: Compound **B24** ^13^C-NMR spectrum.
