# Supplementary material for: Synthesis and Biological Evaluation of New Thiosemicarbazone Derivative Schiff Bases as Monoamine Oxidase Inhibitory Agents
Source: Molecules. 2017 Dec 28;23(1):60. doi: 10.3390/molecules23010060 (PMC6017703; doi:10.3390/molecules23010060)
Supplement: Supplementary file 1 [file molecules-23-00060-s001.pdf]

Supporting Information

Synthesis and Biological Evaluation of New Thiosemicarbazone Derivative Schiff Bases as Monoamine Oxidase Inhibitory Agents

Betül Kaya Çavuşoğlu [1], Begüm Nurpelin Sağlık [1,2], Derya Osmaniye [1,2], Serkan Levent [1,2], Ulviye Acar Çevik [1,2], Abdullah Burak Karaduman [3], Yusuf Özkay [1,2,*] and Zafer Asım Kaplancıklı [1,2]

[1] Department of Pharmaceutical Chemistry, Faculty of Pharmacy, Anadolu University, 26470, Eskişehir, Turkey; betulkaya@anadolu.edu.tr (B.K.Ç.); bnsaglik@anadolu.edu.tr (B.N.S.); dosmaniye@anadolu.edu.tr (D.O.); serkanlevent@anadolu.edu.tr (S.L.); uacar@anadolu.edu.tr (U.A.Ç.); zakaplan@anadolu.edu.tr (Z.A.K.)

[2] Doping and Narcotic Compounds Analysis Laboratory, Faculty of Pharmacy, Anadolu University, 26470, Eskişehir, Turkey

[3] Department of Pharmaceutical Toxicology, Faculty of Pharmacy, Anadolu University, 26470, Eskişehir, Turkey; abkaraduman@anadolu.edu.tr

* Correspondence: yozkay@anadolu.edu.tr; Tel.: +90-222-335-0580 / 3603

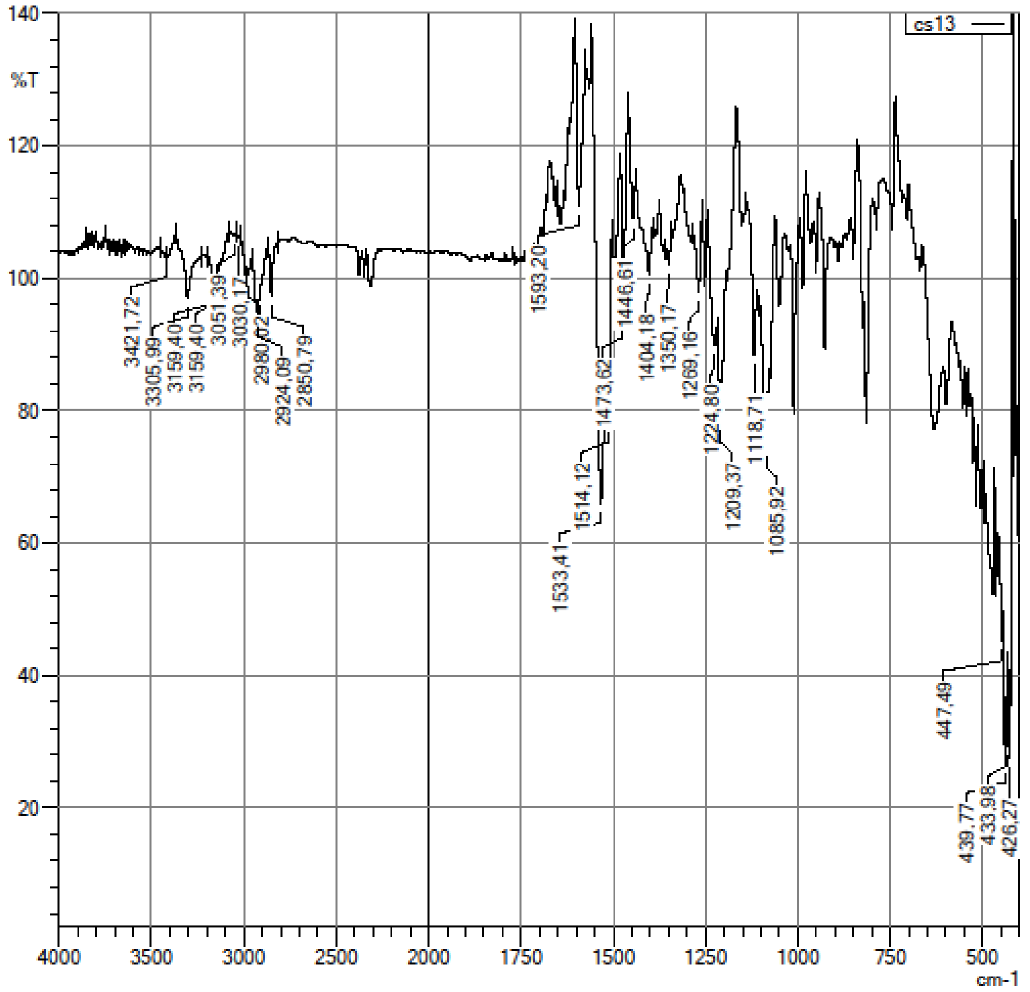

Figure S1. Compound B24 IR spectrum

Data File: C:\LabSolutions\Data\Analiz\BKaya\CS-16-a_31.lcd

Elmt	Val.	Min	Max	Elmt	Val.	Min	Max	Elmt	Val.	Min	Max	Use Adduct
H	1	0	35	O	2	0	5	Cl	1	0	3	H
C	4	0	26	F	1	0	0	Br	1	0	1	
N	3	3	5	S	2	1	2	I	3	0	0	

Error Margin (ppm):	5	DBE Range:	8.0 - 12.0	Electron Ions:	both
HC Ratio:	unlimited	Apply N Rule:	yes	Use MSn Info:	no
Max Isotopes:	3	Isotope RI (%):	1.00	Isotope Res:	10000
MSn Iso RI (%):	10.00	MSn Logic Mode:	AND	Max Results:	500

Event#: 1 MS(E+) Ret. Time : 7.853 -> 7.853 Scan# : 1179 -> 1179

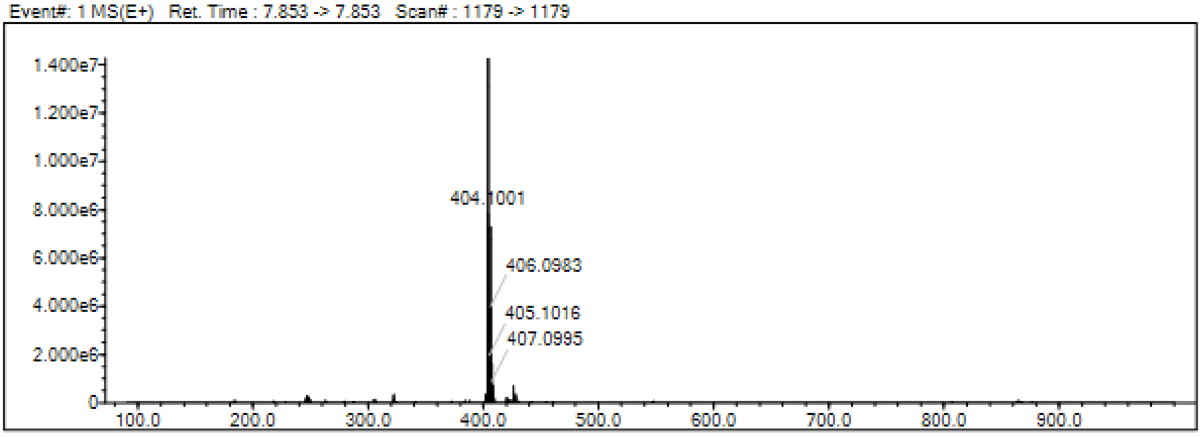

Measured region for 404.1001 m/z

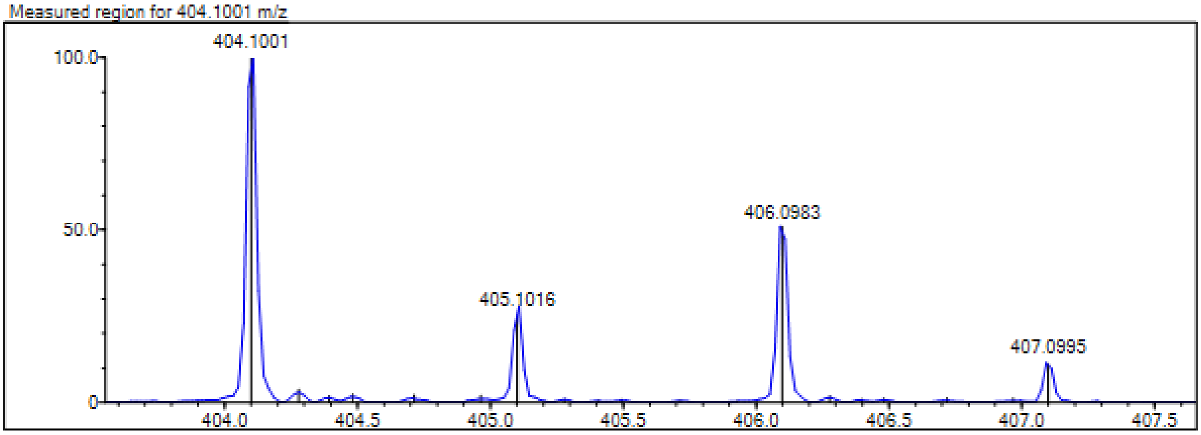

C20 H22 N3 S2 Cl [M+H]+ : Predicted region for 404.1016 m/z

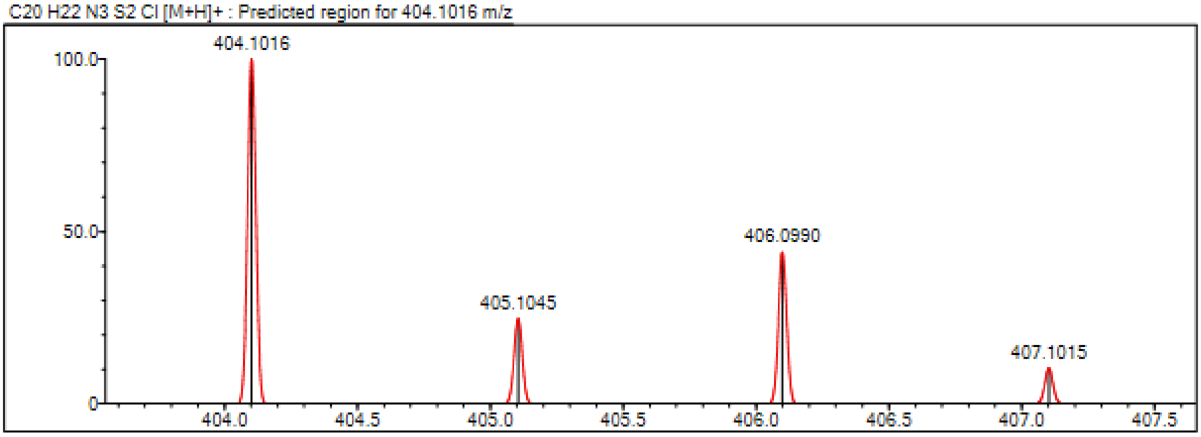

Rank	Score	Formula (M)	Ion	Meas. m/z	Pred. m/z	Df. (mDa)	Df. (ppm)	Iso	DBE
1	84.69	C20 H22 N3 S2 Cl	[M+H]+	404.1001	404.1016	-1.5	-3.71	90.85	11.0

Figure S2. Compound B24 mass spectrum

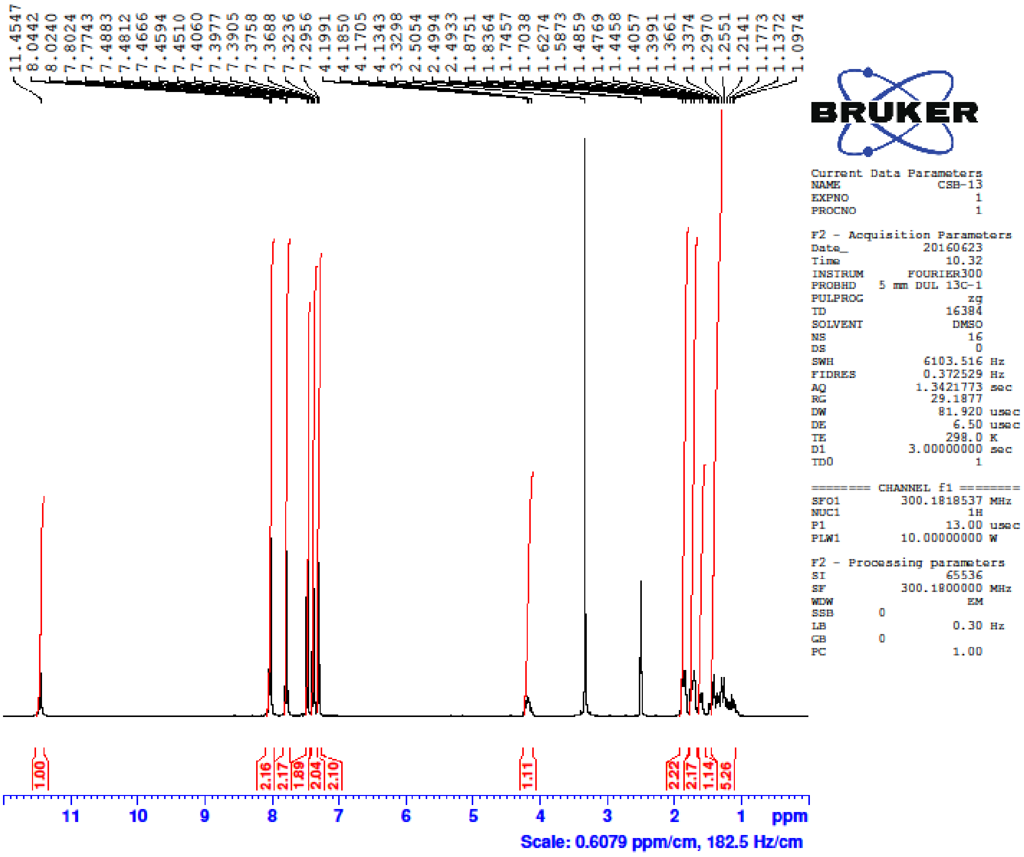

Figure S3. Compound B24 ^{1}H-NMR spectrum

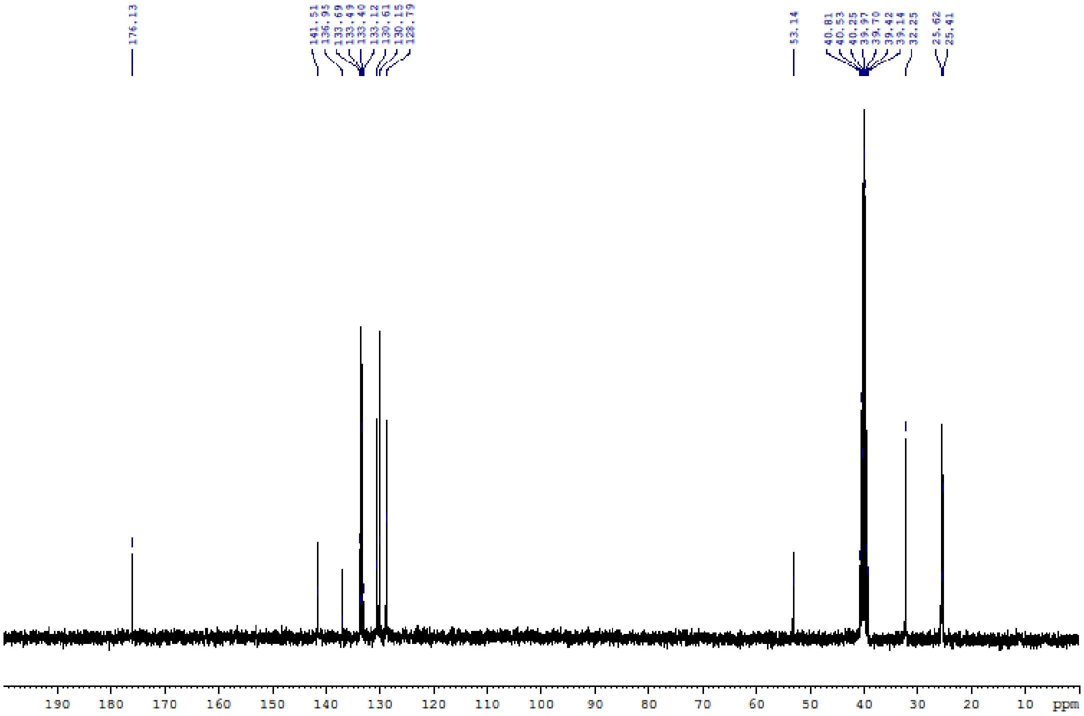

Figure S4. Compound B24 ^{13}C-NMR spectrum
